# Removal of chronic *Mycoplasma ovipneumoniae* carrier ewes eliminates pneumonia in a bighorn sheep population

**DOI:** 10.1002/ece3.6146

**Published:** 2020-03-05

**Authors:** Tyler J. Garwood, Chadwick P. Lehman, Daniel P. Walsh, E. Frances Cassirer, Thomas E. Besser, Jonathan A. Jenks

**Affiliations:** ^1^ Department of Natural Resource Management South Dakota State University Brookings SD USA; ^2^ South Dakota Department of Game Custer SD USA; ^3^ U.S. Geological Survey National Wildlife Health Center Madison WI USA; ^4^ Idaho Department of Fish and Game Lewiston ID USA; ^5^ Department of Veterinary Microbiology and Pathology Washington State University Pullman WA USA

**Keywords:** bighorn sheep, chronic carriage, *Mycoplasma ovipneumoniae*, pathogen persistence, pneumonia, targeted removal, wildlife disease

## Abstract

1. Chronic pathogen carriage is one mechanism that allows diseases to persist in populations. We hypothesized that persistent or recurrent pneumonia in bighorn sheep (*Ovis canadensis*) populations may be caused by chronic carriers of *Mycoplasma ovipneumoniae* (*Mo*). Our experimental approach allowed us to address a conservation need while investigating the role of chronic carriage in disease persistence.
2. We tested our hypothesis in two bighorn sheep populations in South Dakota, USA. We identified and removed *Mo* chronic carriers from the Custer State Park (treatment) population. Simultaneously, we identified carriers but did not remove them from the Rapid City population (control). We predicted removal would result in decreased pneumonia, mortality, and *Mo* prevalence. Both population ranges had similar habitat and predator communities but were sufficiently isolated to preclude intermixing.
3. We classified chronic carriers as adults that consistently tested positive for *Mo* carriage over a 20‐month sampling period (*n* = 2 in the treatment population; *n* = 2 in control population).
4. We failed to detect *Mo* or pneumonia in the treatment population after chronic carrier removal, while both remained in the control. Mortality hazard for lambs was reduced by 72% in the treatment population relative to the control (CI = 36%, 91%). There was also a 41% reduction in adult mortality hazard attributable to the treatment, although this was not statistically significant (CI = 82% reduction, 34% increase).
5. *Synthesis and Applications*: These results support the hypothesis that *Mo* is a primary causative agent of persistent or recurrent respiratory disease in bighorn sheep populations and can be maintained by a few chronic carriers. Our findings provide direction for future research and management actions aimed at controlling pneumonia in wild sheep and may apply to other diseases.

## Graphical abstract

In this experimental study, we removed chronic shedders of *Mycoplasma ovipneumoniae* from a pneumonic bighorn sheep population and followed adult and juvenile survival in both the population and a nearby control population. The treatment population had significantly higher survival across age classes and no pneumonia or *M. ovipneumoniae*, whereas the control population suffered high mortality rates due to pneumonia. Pneumonia mortality is a widespread and persistent threat to bighorn sheep populations, and this is the first management action that has successfully removed pneumonia from a herd.

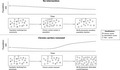

## 1 INTRODUCTION

Heterogeneity in infectiousness of individual hosts may dramatically affect pathogen transmission, as illustrated by the role of human superspreaders in the emergence of the severe acute respiratory syndrome‐associated coronavirus (SARS‐CoV), a zoonotic virus responsible for an acute human epidemic during 2002–2003 (Lloyd‐Smith, Schreiber, Kopp, & Getz, 2005; Woolhouse et al., 1997). Less is known about the importance of individual variation in infectiousness among diseases associated with chronically carried bacterial pathogens. Chronic carriage/shedding is common features of several pathogenic bacteria, so understanding infection risks posed by chronic carrier individuals is relevant to management of infectious diseases across human, domestic animal, and wildlife health sectors (Buhnerkempe et al., 2017; Coyne et al., 2006; Wertheim et al., 2005).

Bighorn sheep (*Ovis canadensis*) populations declined precipitously following the mid‐1800s (Buechner, 1960), and bacterial pneumonia is a primary impediment to recovery (Cassirer et al., 2018). Bighorn pneumonia frequently manifests as an initial all‐age outbreak causing 5%–100% mortality (Cassirer et al., 2018; Enk, Picton, & Williams, 2001; Spraker, Hibler, Schoonveld, & Adney, 1984), followed by annual epizootics among juveniles and sporadic pneumonia mortality among adults (Cassirer et al., 2013; Smith, Jenks, Grovenburg, & Klaver, 2014). Previous unsuccessful attempts to control pneumonia targeted *Pasteurellaceae* (*Bibersteinia trehalosi*, *Mannheimia haemolytica,* leukotoxigenic *Pasteurella*) and parasites (*Protostongylus* sp.) (Dassanayake et al., 2009; Foreyt, Snipes, & Kasten, 1994; Miller et al., 2000). While *Mycoplasma ovipneumoniae* (*Mo*) is more strongly associated with pneumonia than previously targeted pathogens (Besser et al., 2008, 2013), no vaccines or efficacious antimicrobial treatments for *Mo* currently exist*.* Interventions for any pathogen(s) are also constrained by inaccessible terrain, as well as the complex movement patterns of bighorn sheep (Cassirer et al., 2018; George, Martin, Lukacs, & Miller, 2008). Thus, effective management strategies for addressing bighorn sheep pneumonia are currently lacking and clearly needed to conserve the species.

**Figure 1:**
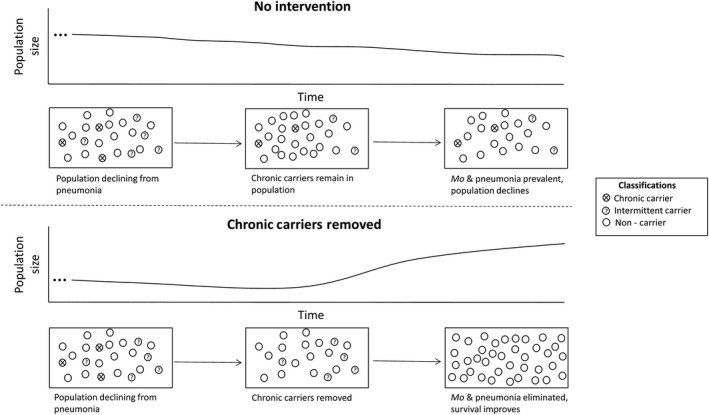
A conceptual depiction of our experiment, where chronic carriers of *Mycoplasma ovipneumoniae* are identified in two populations, but only removed in one. Bighorn sheep can be classified as chronic carriers, intermittent carriers, and noncarriers; only chronic carriers need to be removed under our operating hypothesis. If chronic carriers are removed, the population should rebound as lamb recruitment improves. Without intervention, the population will continue to decline indefinitely due to low lamb recruitment

**Figure 2:**
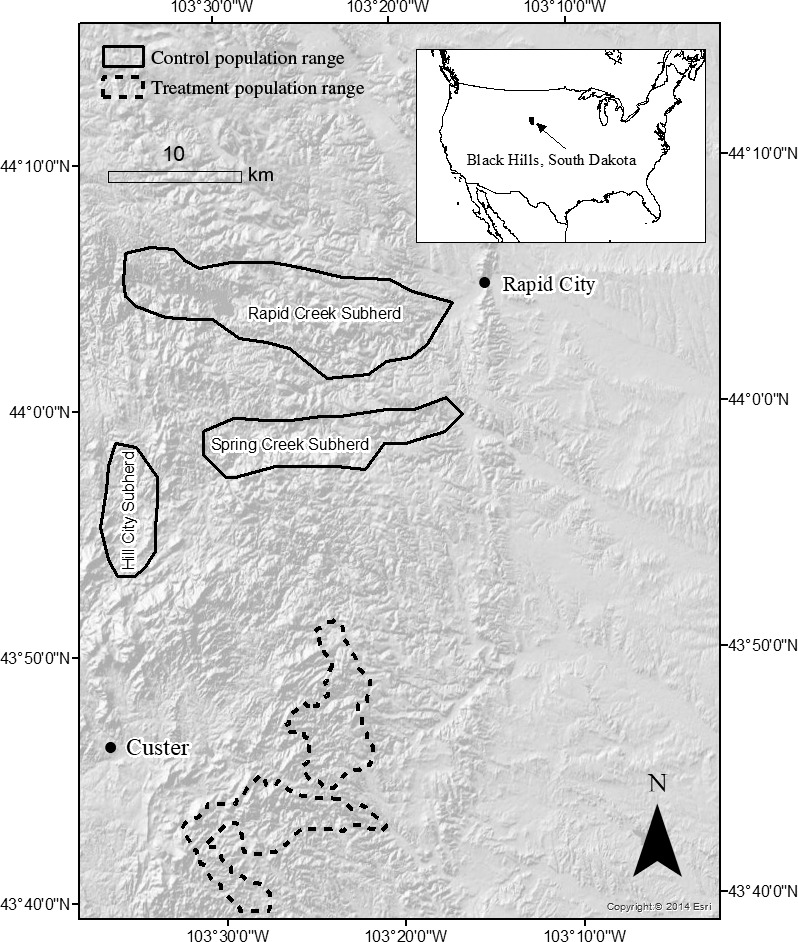
Ranges of study populations of bighorn sheep in the Black Hills, South Dakota, USA, 2014–2018

Bighorn sheep herds with clinical signs of respiratory disease often demonstrate widespread *Mo* exposure (median seroprevalence = 67%) but lower carriage prevalence (median = 22%; Cassirer et al., 2018). Plowright et al. (2017) found that about half of bighorn sheep testing positive for *Mo* consistently tested positive, implying chronic carriage. We therefore hypothesized that pneumonia could be maintained in bighorn sheep populations by a few identifiable, chronic carrier individuals (Figure 1). We tested the hypothesis in free‐ranging bighorn sheep populations in the Black Hills of South Dakota, USA: the Custer State Park treatment population and the Rapid City control population (Figure 2). Our objectives were to determine whether removing chronic carrier individuals (a) reduces the prevalence of *Mo*, (b) reduces pneumonia‐related mortality, and (c) reduces overall mortality.

## 2 MATERIALS AND METHODS

### 2.1 Study area

We conducted our study in the South Dakota Black Hills from August 2014 to May 2018. This study site includes elevations of 972–2,207 m (Brown & Sieg, 2016), average annual precipitation (Rapid City) of 11.3 cm rainfall and 29.6 cm snow, and temperatures of −30 to 41**°**C, with an average high of 17**°**C and an average low of 1°C (National Oceanic & Atmospheric Administration, 2018).

The treatment population inhabited a 28,733‐ha state park in Custer County (Figure 2; 43°46′19″ N, 103°23′16″ W). Canyons and rocky outcroppings characterized bighorn sheep habitat. An all‐age die‐off occurred in 2004, and pneumonia deaths persisted through 2016 (South Dakota Department of Game, Fish, & Parks, 2018). When we began building *Mo* carriage profiles in August 2014, the treatment population numbered 14 ewes, 8 rams, and 3 lambs. Lamb:ewe ratios averaged 0.205 before chronic carrier removal (2006–2015), and pneumonia deaths were documented among lambs through the autumn of 2015 (South Dakota Department of Game, Fish and Parks, unpublished data).

The control population inhabited public and private land in Pennington County and contained three subherds that rarely interacted (Rapid Creek, Spring Creek, and Hill City, Figure 2; 44°04′37″ N, 103°21’16” W). This population utilized canyons for summer range and residential lawns during winter (Smith, Grovenburg, Monteith, & Jenks, 2015). An all‐age die‐off occurred in 2009, and pneumonia remained a major mortality source (Smith, Jenks, et al., 2014). The population numbered approximately 45 ewes, 20 rams, and 5 lambs in 2016. South Dakota Game, Fish, and Parks annually collect lamb:ewe ratio data November–December, and lamb:ewe ratios in the control population averaged 0.18 from 2007 to 2015 (South Dakota Department of Game, Fish, & Parks, 2018). We did not observe movement between populations during the study.

### 2.2 Radio‐marking, data collection, pathogen detection, and strain typing in adults

We captured bighorn sheep via chemical immobilization (BAM; 0.43 mg/kg butorphanol, 0.29 mg/kg, azaperone, 0.17 mg/kg medetomidine, Wildlife Pharmaceuticals) delivered through dart injection or by aerial net‐gunning (Quicksilver Air, Inc. and Hells Canyon Helicopters). We fitted sheep with very high‐frequency (VHF) collars (M252OB; Advanced Telemetry Systems) with mortality sensors, which were activated if the collar was motionless for ≥8 hr. After confirming pregnancy with ultrasonography (E.I. Medical Imaging), we fitted ewes with VHF vaginal implant transmitters (VITs; M3900; Advanced Telemetry Systems; Smith, Walsh, et al., 2014).

During capture, we collected information on sex, age, *Mo* carrier status and antibodies, and presence of other pathogens. We aged individuals up to 3.5 years old using tooth eruption (Valdez & Krausman, 1999) and classified adults >3.5 years old as a single age group. We collected *Mo* mucosal samples via three swabs, which were consecutively inserted deep into each of the nares and rotated around the cavity wall during removal (Drew et al., 2014). We returned two swabs to their sheath and immersed one in Tryptic Soy Broth with 15% Glycerol (Hardy Diagnostics; Butler et al., 2017). To sample for other aerobic bacteria that could contribute to respiratory disease, we rotated 3 swabs along each tonsillar crypt and stored them similarly to nasal swabs. We collected blood for serum, held all samples at 4°C, and shipped them on ice to the Washington Animal Disease Diagnostic Lab (WADDL) or Dr. Thomas Besser's lab (Washington State University).

We used real‐time polymerase chain reaction (RT‐PCR) to detect *Mo* on nasal swabs (Ziegler et al., 2014). Samples were positive if fluorescence generation exceeded the threshold before the 36th RT‐PCR cycle, indeterminate if between the 36th and 40th cycles, and negative if not exceeded through 40 cycles. We determined serum *Mo* antibody presence by competitive enzyme‐linked immunosorbent assay (c‐ELISA; Ziegler et al., 2014). We documented other pathogens on swabs through aerobic culture and PCR (Besser et al., 2008).

We determined *Mo* strain type using genomic DNA extracted from *M. ovipneumoniae* broth cultures or swabs using DNeasy blood and tissue kits (Qiagen Inc.), following manufacturer's instructions, or PCR‐positive DNA extracts obtained directly from WADDL. *Mycoplasma ovipneumoniae* extracts were genotyped using a multi‐locus sequence typing approach that targets four loci. The targeted loci are partial DNA sequences from the 16S‐23S intergenic spacer region (IGS), the small ribosomal subunit (16S), and housekeeping genes encoding RNA polymerase B (*rpoB*) and gyrase B (*gyrB*). Protocols and primers for PCR amplification of these loci were described previously (Cassirer, Manlove, Plowright, & Besser 2017). Commercial service laboratories (Amplicon Express and Eurofins Genomics) conducted bidirectional Sanger DNA sequencing of amplified PCR products using the same primers used in PCR reactions.

### 2.3 Chronic carrier identification and experimental removal

We began *Mo* testing in the treatment population in August 2014. We obtained ≥2 tests from every adult alive in the population before or shortly after chronic carrier removal (Table 1 and Table S1). We classified individuals as chronic carriers (consistently positive), intermittent carriers (negative and positive tests), or noncarriers (all negative tests). We immobilized and relocated all chronic carriers from the treatment population to South Dakota State University (SDSU). Postremoval, we tested most individuals in both populations annually (Table S2 and S3).

**Table 1:** Summary of bighorn sheep pathogen testing results and survival estimates in the treatment and control populations, 1 August 2014–8 April 2018. Individuals in the treatment population that had at least two tests and always tested positive after the first positive test were classified as chronic carriers and removed 13 March 2016. Proportions of chronic carriers are based only on individuals sampled at least two times. *Mycoplasma ovipneumoniae* (*Mo*) carriage was detected through real‐time polymerase chain reaction (RT‐PCR), antibody status was determined through enzyme‐linked immunosorbent assay, and the presence of other pathogens was ascertained through culture and PCR. Survival estimates are presented with their respective 95% credible intervals (CI)

Population	*Mo*‐positive tests	Definitive chronic carriers	Intermittent carriers	Positive for *Mo* antibodies	Other pathogens detected	Adult survival estimate	Lamb survival estimate
Treatment (pre‐removal)	8 of 60 (13%)	2 of 22 (9%)	1 of 22 (5%)	18 of 24 (75%)	*Bibersteinia trehalosi*, *Trueperella pyogenes*, *Mannheimia* sp., Leukotoxigenic *Pasteurella*	Not estimated	Not estimated
Treatment (postremoval)	0 of 35 (0%)	0 of 9 (0%)	0 of 9 (0%)	10 of 20 (50)%	*Bibersteinia trehalosi*, *Trueperella pyogenes*, *Mannheimia* sp., *Mannheimia haemolytica*, *Pastuerella* sp.	94% CI = 77%–99%	77% CI = 26%–96%
Control	38 of 80 (48%)	2 of 20 (10%)	11 of 20 (55%)	49 of 57 (85%)	*Bibersteinia trehalosi*, *Trueperella pyogenes*, *Mannheimia* sp., *Mannheimia haemolytica*, *Mannheimia glucosida*, Leukotoxigenic *Pasteurella*	88% CI = 82%–92%	35% CI = 12%–62%

### 2.4 Lamb capture

We monitored VIT radio signals daily using handheld directional radio telemetry (Telonics, Inc.) starting 15 April 2016 and 2017. We located the dam to verify lamb presence when a VIT was expelled. Budgetary constraints prevented VIT implantation in some pregnant ewes in the control population, so we visually monitored females without VITs along with ewes that expelled their VITs prematurely. If we observed a lamb, we attempted to hand capture it. We immobilized lambs we failed to capture as neonates at 2–6 months old. We weighed captured lambs, determined sex, and fitted them with expandable VHF collars (M4200; Advanced Telemetry Systems). We wore latex gloves and minimized handling of neonates to avoid abandonment (Smith, Walsh, et al., 2014). The SDSU Institutional Animal Care and Use Committee approved capture and handling procedures (Approval number 16‐00A).

### 2.5 Survival monitoring

We monitored adult collars for mortality signals 2 times/week from 13 March 2016 to 1 May 2018 and lamb collars daily from capture until 20 November; thereafter, we monitored lambs similarly to adults. When a collar indicated mortality, we located the carcass and examined the site for predator presence (scat, tracks, scrapes) and the cadaver for caching, hemorrhaging, and skeletal disarticulation (Stonehouse, Anderson, Peterson, & Collins, 2016). We performed necropsies on adults in the field and shipped swabs to WADDL for pathogen testing. When possible, we shipped lamb cadavers to WADDL for necropsy.

Cause of death was sometimes uncertain; therefore, we used the knowledge gained from necropsies, evidence at the site, and behavioral observations to assign the likelihood of each individual's cause of death. Cause‐specific mortality categories for lambs and adults were “predation,” “pneumonia,” and “other,” and, in addition for adults only, “human‐caused.” We created a probability for each category, and the resulting vector of prior predictive probabilities summed to one for each individual. If cause of death was certain, the vector contained a single, nonzero entry for the appropriate cause‐of‐death category (Walsh, Norton, Storm, Van Deelen, & Heisey, 2018). As an example of this process, a carcass might be scavenged shortly after dying, and movement of the collar could delay detection of the mortality by a week. The lack of disarticulation might rule out predation, but the carcass's desiccated state would make it difficult to tell whether pneumonia or another internal malady caused death. If we had previous behavioral evidence that indicated pneumonia, in this example we would assign a 70% predictive probability to “pneumonia,” 30% to “other,” and 0% to all other categories.

### 2.6 Survival analysis

#### 2.6.1 Adult Covariates

We modeled weekly survival rates, incorporating covariates for treatment, strain type exposure (Cassirer et al., 2017), testing PCR positive for *Mo*, biological year (Gaillard, Festa‐Bianchet, Yoccoz, Loison, & Toigo, 2000), sex (Jorgenson, Festa‐Bianchet, Gaillard, & Wishart, 1997), and age (Loison, Festa‐Bianchet, Gaillard, Jorgenson, & Jullien, 1999) as factors affecting survival. We coded the treatment, sex, and an individual's positive test effects as binary variables (treatment population = 1, control population = 0; males = 1, females = 0; ≥1 positive = 1, no positives = 0). We treated an individual's age as a time‐varying, categorical variable separated into 3 groups (0 = 1–2 years old, 1 = 2–3 years old, 2 = 3+ years old). Individuals advanced in age class on 22 May to correspond with peak lambing time in our study area. For each individual, the year effect was a time‐varying, categorical variable spanning 2016–2018, with each week assigned to the appropriate calendar year. Based on strain typing data, adult bighorns in the Spring Creek subherd were exposed to an introduced strain sometime between 14 November 2016 and 13 December 2016. We therefore set time of exposure to the introduced strain in Spring Creek at the midpoint between those dates and coded strain type exposure as binary and time‐varying (exposure to introduced strain BHS‐043 = 1, exposure to resident strain BHS‐058 = 0; Kamath, Manlove, Cassirer, Cross, & Besser, 2019). All adults outside the Spring Creek subherd were modeled as only exposed to the resident strain (BHS‐058) for the duration of the study.

#### 2.6.2 Adult a priori models

Our adult global model calculated log unit cumulative hazard as ln(*Λ_i,j_*) = *γ* + *β*_treatment_ × treatment_i_ + *β*_positive test_ × positive test_i_ + *β*_strain_ [strain type*_ij_*] + *β*_year_ [year*_ij_*] + *β*_age_ [age*_ij_*] + *β*_sex_ × sex_i_ + *ρ_j_*, where *γ* was the baseline log unit cumulative hazard rate. For the *i* ^th^ individual during the *j* ^th^ week, we denoted *β*_treatment_ as the treatment effect, *β*_positive test_ as the positive test effect, *β*_strain_ as the strain type effect, *β*_year_ as the year effect, *β*_age_ as the individual's age effect, and *β*_sex_ as an individual's sex effect. We signified the week effect with *ρ_j_*.

#### 2.6.3 Lamb covariates

We modeled daily survival rates, incorporating treatment (i.e., population identity), year (Gaillard et al., 2000), strain type exposure (Cassirer et al., 2017), sex (Rioux‐Paquette, Festa‐Bianchet, & Coltman, 2011), birth timing (Feder, Martin, Festa‐Bianchet, Berube, & Jorgenson, 2008), and birth weight (Festa‐Bianchet, Jorgenson, Berube, Portier, & Wishart, 1997) as factors that might affect survival to 0.5 years old. We coded treatment and individual sex effects as with adults. We designated year as a binary variable (2016 = 0, 2017 = 1) and birth timing consisted of three groups: lambs born within ± 3 days of the median date of lamb births in a given year, those born >3 days before the peak period, and those born >3 days after the peak period (Smith, Jenks, et al., 2014). We coded strain type exposure as categorical (exposure to introduced strain = 2, exposure to resident strain = 1, exposure to neither = 0). Based on strain typing data, lambs in the Spring Creek subherd were exposed to the introduced strain in 2017, and the resident strain in 2016. All lambs in Rapid Creek and Hill City subherds were classified as only exposed to the resident strain for the duration of the study. Since we removed all carriers of *Mo* in the treatment population in January 2016 and there were no *Mo*‐positive tests postremoval, we classified treatment lambs as being exposed to neither strain for the entire study. Birth weight was measured to the nearest 0.10 kg. For late‐caught lambs, we imputed missing birth weight and birth timing values using the empirical distributions of these variables for lambs caught as neonates (Gelman et al., 2014).

#### 2.6.4 Lamb a priori models

Our global model calculated daily log unit cumulative hazard as ln(*Λ_i,j_*) = *γ* + *β*_treatment_ × treatment_i_ + *β*_strain_ [strain type*_i_*] + *β*_year_ × year*_i_* + *β*_sex_ × sex_i_ + *β*_birth timing_ [birth timing_i_] + *β*_birth weight_ × birth weight_i_ + *ρ_j_*. The *γ*, *β*_treatment_, and *β*_sex_ parameters were included in the model in the same way as in the adult model. For the *i* ^th^ individual during the *j* ^th^ day, we denoted the year effect as *β*_year_, *β*_strain_ as the strain type exposure effect, *β*_birth timing_ as the birth timing effect, and *β*_birth weight_ as the birth weight effect. We signified the day effect with *ρ_j_*.

#### 2.6.5 Analysis framework

Implementing a Bayesian time‐to‐event framework through the Nimble package (de Valpine et al., 2017) in Program R (R Core Team, 2018), we performed separate survival analyses for lambs and adults to fit models to both survival datasets (Walsh et al., 2018). Our framework first calculated hazard of dying irrespective of cause of death (Cross et al., 2015) using weakly informative truncated normal priors on the baseline log unit cumulative hazards. We assumed a mean annual adult survival of 86% and a 95% probability of lying between 20% and 95% (*y* ~ Normal [−3.745, *σ* ^2^ = 0.29] *T*[−9.5, −2]; Loison et al., 1999), and 50% mean annual lamb survival with a 95% probability of lying between 10% and 80% (*y* ~ Normal [−6.26, *σ* ^2^ = 0.33] *T*[−8, −1]; Parr, Smith, Jenks, & Thompson, 2018). We specified an intrinsically conditional autoregressive prior (Cressie & Wikle, 2011) for the week effect in adults and day effect in lambs (*ρ_j_*) to provide temporal smoothing across unit log hazard estimates. Specifically, we assigned a prior with a uniform distribution (*ρ_l_* ~ Uniform [−0.5, 0.5]) for the first week or day effect and specified the effect for the *j* ^th^ week or day as *ρ_j_* ~ Normal (*ρ_j_*_−1_, *σ* ^2^ = 1/*τ*). Lastly, we specified the prior for the precision parameter as: (*τ* ~ Gamma [1, 1]). Priors on covariate effects were diffuse (*β_x_* ~ Normal [0, *σ* ^2^ = 100]).

We then calculated cause‐specific mortality while incorporating observer uncertainty into parameter estimation (Walsh et al., 2018). Specifically, we treated the true cause of death for each individual as a latent, unknown variable with an assigned vector of prior predictive probabilities. As described above, these priors specified the observer's belief that each cause of death was the true cause of death given their assessment of the available evidence (Table S4). We imputed the true cause of death, using a data augmentation approach that generated a cause of death at each Markov chain Monte Carlo (MCMC) iteration (Gelman et al., 2014), based on a categorical distribution with a parameter vector equal to the prior predictive probability vector specified for that individual. Using random starting values, we ran three MCMC chains for 100,000 iterations and removed the first 10,000 repetitions for burn‐in. We looked for evidence of nonconvergence of the chains via graphical checks, and none was observed for any of the following analyses.

We calculated Watanabe‐Akaike Information Criteria (WAIC) from each model to identify models that best described the evidence in the data (Gelman et al., 2014). We considered models differing by ≤2 WAIC as alternatives to the top ranked model but preferred the simplest model (Burnham & Anderson, 2002). We based our conclusions on parameter estimates from the best model.

## 3 RESULTS

### 3.1 Adults

#### 3.1.1 Collaring effort

We radio‐collared 86 adult bighorn sheep between 1 August 2014 and 1 May 2018:29 in the treatment (10 rams, 19 ewes) and 57 (19 rams, 38 ewes) in the control population. This constituted 100% and ~90% of adults in the respective populations by the study's end. Categorized by age at the end of the study, we collared 16 (9 treatment, 7 control) 1–2 year olds, 4 (2 treatment, 2 control) 2–3 year olds, 10 (4 treatment, 6 control) 3–4 year olds, and 56 (14 treatment, 42 control) ≥4 year olds.

#### 3.1.2 Chronic carrier identification and removal in the treatment population

We tested all treatment population adults that survived long enough to be tested (7 rams, 17 ewes) for *Mo* over 60 sampling events (Table 1). Fifty‐one (85%) swabs tested negative with RT‐PCR, 1 (2%) tested indeterminate, and 8 (13%) tested positive. Two females were classified as chronic carriers of the BH‐058 strain (*n* = 7 positive tests). The other adult that tested positive subsequently tested negative and died before experimental manipulation. We classified all other individuals tested twice as noncarriers (*n* = 19 individuals). We removed the two chronic carriers on 13 March 2016.

#### 3.1.3 Postremoval pathogen testing in the treatment population

After removal of chronic carriers, we did not detect *Mo* in 35 samples (26 negative and 9 indeterminate) collected from 26 individuals (9 males, 17 females) in the treatment population (Table 1 and Table S3). We collected serum from 20 individuals, and antibodies were detected in 10 (50%); 2 (10%) tested indeterminate, and 8 (40%) tested negative. We detected *Trueperella pyogenes* in 4 bighorn sheep (15%), *B. trehalosi* in 8 (31%), *Mannheimia* sp. in 4 (15%), *M. haemolytica* in 1 (4%), and *Pasteurella* sp. in 1 (4%).

#### 3.1.4 Pathogen testing and strain typing in the control population

Between 1 January 2016 and 1 May 2018, we collected 80 samples from 57 control population bighorn sheep (Table 1 and Table S2). We obtained 38 *Mo*‐positive samples (48%), 9 indeterminate samples (11%) and 32 negative samples (41%). We were able to strain type 36 of the 38 positive samples. We found that 24 samples were the resident BHS‐058 strain also detected in the treatment population, but 11 samples were typed as BHS‐043 (introduced). A single detection of a unique strain in Spring Creek in February 2016 may be attributable to a laboratory cross‐contamination error or a spillover that faded out before onward transmission. All introduced strain samples were collected in the Spring Creek subherd on or after 13 December 2016. Excluding indeterminate test results, we identified 2 chronic carriers (of the resident strain), 11 intermittent carriers (resident or introduced strain), and 3 noncarriers. We could not classify carrier status of 37 individuals that were tested only once (21 with 1 positive test, 16 with 1 negative test). Antibodies to *Mo* were detected in 48 of 51 bighorns (94%); 1 (2%) tested indeterminate, and 2 (4%) tested negative. We lacked serum from 6 individuals. We documented *T. pyogenes* (*n* = 25, 44% of control population), *B. trehalosi* (*n* = 22, 39%), *Mannheimia* sp. (*n* = 2, 4%), *M. haemolytica* (*n* = 1, 2%), *Mannheimia glucosida* (*n* = 1, 2%)*,* and leukotoxigenic *Pasteurella* (*n* = 1, 2%).

#### 3.1.5 Survival analysis

**Figure 3:**
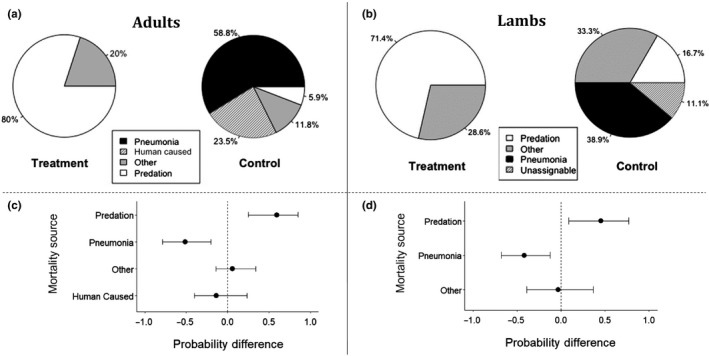
A comparison of mortality sources for bighorn sheep between herds after chronic carrier removal in the treatment population on 13 March 2016. Panels [a] and [b] show cause‐specific mortality sources based on the most likely cause of death assigned in the field for adults and lambs, respectively. Panels [c] and [d] show the estimated differences in cause‐specific probabilities between the populations based on the survival analysis for adults and lambs, respectively. Estimates for the treatment population are given relative to the control population, which is signified by the dotted line at 0. Credible intervals are 95%

We monitored survival of 86 radio‐collared adults 13 March 2016–1 May 2018. We documented 5 mortalities (2 males, 3 females, 17% of adults) in the treatment population and 17 (6 males, 11 females, 30% of adults) in the control population (Figure 3a). Our best survival model was ln(*Λ_ij_*) = *γ* + *β*_treatment_ × treatment*_i_* + *ρ_j_*, which we used to calculate log weekly cumulative mortality hazard estimates (*w* = 0.31, Table S5). The model indicated chronic carrier removal reduced adult hazard by 41%, but was not statistically significant with the 95% credible interval (CI) including zero (CI = 82% reduction, 34% increase; Table 2, Figure S1). An analysis that excluded the Spring Creek subherd, which was exposed to multiple *Mo* strains, produced similar estimates (hazard reduction = 48%, CI = 85% reduction, 20% increase). This corresponds to a 94% annual survival rate (CI = 77%, 99%) in the treatment population and 88% (CI = 82%, 92%) in the control population (Table 1).

**Table 2:** Hazard ratios corresponding with the treatment effect for lambs and adults. Ratios are given relative to the control population

Age group	Hazard ratio	95% credible interval
Adults	0.59	0.18, 1.34
Lambs	0.28	0.09, 0.64

Hazard of pneumonia‐induced mortality for adults in the treatment population was significantly lower than in the control population (Probability difference = −52%, CI = −78%, −15%; Figure 3c). No pneumonia was detected in the treatment population, and the probability of pneumonia‐induced adult mortality was lower (10%, CI = 0%, 41%) than in the control herd (61%, CI = 36%, 84%). Conversely, predation was more likely to be assigned as cause of death in the treatment population (61%, CI = 22%, 92%) than in the control (10%, CI = 2%, 27%; probability difference = 51%, CI = 13%, 83%).

### 3.2 Lambs

We analyzed mortality data collected 1 May–1 November in 2016–2017 on 43 lambs. We captured 37 neonatal lambs (2016:5 treatment, 15 control; 2017:10 treatment, 7 control) and immobilized another six lambs 2–6 months postparturition (2016:4 treatment, 2 control). Twenty‐seven (60%) lambs died 1 May–1 November, before 6 months of age. We excluded one lamb that was seriously injured during capture and another that was caught during late‐stage pneumonia. Of the 25 mortalities analyzed, seven (5 males, 2 females) occurred in the treatment and 18 (6 males, 12 females) in the control population (Figure 3b). This constituted 35% of treatment population lambs and 69% of control lambs. Lamb:ewe ratios after chronic carrier removal in the treatment population was 0.48, whereas the control population exhibited a ratio of 0.08 over the same time period (2016–2018).

Our best lamb survival model was ln(*Λ_ij_*) = *γ* + *β*_treatment_ × treatment_i_ + *β*_year_ × year*_i_* + *ρ_j_* (*w_i_* = 0.64; Table S6). Lamb birth weight (mean = 5.5 kg, *SE* = 0.08, *n* = 37), sex, and birth timing were not supported as predictors of survival. We calculated log daily cumulative hazard measurements based on this model (Figure S2) and determined that chronic carrier removal had a negative effect on daily lamb hazard (72% reduction in the treatment population, CI = 36%, 91%; Table 2). This corresponds with a 77% annual survival rate (CI = 26%, 96%) in the treatment population and 35% (CI = 12%, 62%) in the control (Table 1). We observed higher lamb mortality in 2017 than in 2016 (*β*_year_ = 1.15, CI = 0.29, 2.02).

Pneumonia‐caused mortality was significantly less likely in the treatment population (probability = 6%, CI = 0%, 29%) than in the control population (probability = 48%, CI = 24%, 73%), with a probability difference of −37% (CI = −59%, −1%) (Figure 3d). Lambs that died had a 63% probability of dying from predation in the treatment population (CI = 28%, 90%), which was significantly higher than the control population (17%, CI = 4%, 38%; probability difference = 39%, CI = 3%, 77%).

## 4 DISCUSSION

We found that pneumonia can be maintained in bighorn sheep populations by a few individuals chronically carrying *Mo*. After removing these individuals, we detected no deaths attributable to pneumonia and 76% of lambs survived to 6 months of age, similar to other healthy populations in our study region (37.5%–90%; Parr et al., 2018; Zimmerman, 2008). In contrast, we detected pneumonia‐induced mortality in adults and juveniles in a control population where *Mo* carriers remained. Average lamb survival to 6 months in the control population was 35%, which is similar to other unhealthy populations (0%–60% of collared lambs surviving; Cassirer et al., 2018; Grigg et al., 2017; Smith, Jenks, et al., 2014). We detected other pneumonia‐associated pathogens in both populations, but their presence failed to induce pneumonia in adults or lambs in the absence of *Mo* (Table 1). This finding makes physiological sense: while other pathogens contribute to disease (Besser et al., 2013), *Mo* appears necessary, by disrupting mucociliary clearance, for bighorn sheep to establish lung infections (Cassirer et al., 2018; Niang et al., 1998). Although leukotoxigenic *Pasteurella* was detected in the treatment population before removal and not afterward, the three individuals harboring it were not removed (Table S1 and Table S3). Our results indicate that *Mo* is warranted as a focal pathogen in efforts to eliminate pneumonia in wild sheep populations.

We found 11 intermittent carriers in our control population but none in our treatment population (Table 1). Strain typing of samples from intermittent carriers in the control population revealed that eight changed from noncarriers to carriers when infected with a strain previously found in bighorn sheep in Deadwood, South Dakota and western Nebraska (Kamath et al., 2019). These individuals therefore were not intermittently carrying a single strain of *Mo*. More likely, they lacked strain‐specific immunity and were acutely carrying the introduced strain of *Mo* (Cassirer et al., 2017). Importantly, they were not contributing to the persistence of the original strain. The role of intermittent carriers in population‐level dynamics of *Mo* is not known (Plowright et al., 2017); however, our assumption is that chronic carriers are necessary for persistence. Our study indicates that in some cases intermittent carriage can be attributed to the introduction of a new strain type. Future studies could consider invasion of new strains as a potential factor influencing Mo carriage patterns.

Although our study demonstrates improved lamb survival resulting from removing chronic carriers, the impact on adult survival is not as pronounced. We detected a statistically significant reduction in pneumonia‐induced adult mortality in the treatment population relative to the control, but other sources of mortality in the treatment population offset this effect. For adults, our best survival model included a treatment effect and was significantly better (i.e., ≥2 ΔWAIC) than models lacking this effect; however, the effect was not statistically significant. This finding may indicate that the treatment effect was biologically important, but a larger sample size is needed to assess statistical significance given the overall high survival rates of both the treatment and control populations. Previous studies found that adult survival generally rebounded to or above previous levels in the years following all‐age die‐offs (Manlove, Cassirer, Cross, Plowright, & Hudson, 2016; Plowright et al., 2013). Clarifying the impact of chronic carrier removal on adult survival in future studies would improve our understanding of the long‐term impacts of pneumonia on adults and inform population viability analyses of pneumonic populations.

The introduction of a new *Mo* strain type in one subherd of the control population toward the end of the first year of the study also complicated our assessment of the treatment effect. We attempted to control for this complication by explicitly analyzing the impact of this introduction on survival, and our modeling efforts demonstrated the strain effect was statistically insignificant relative to the treatment effect, and did not appear in any competing model. Therefore, strain introduction did not change our conclusions about the impact of chronic carrier removal in *Mo*‐affected populations.

It remains unclear which host factors contribute to chronic carriage and how to minimize the number of tests necessary to identify chronic carriers. Plowright et al. (2017) found that age and homozygosity at a specific locus were associated with persistent carriage of *Mo* in bighorn sheep, suggesting possible host factors that might contribute to variation in infectious period and carriage rates among populations. Coinfections, underlying disease, and high exposure frequency and/or dose may also shape host resistance to infection and increase likelihood of chronic or intermittent carriage (Fox et al., 2015). Chronic carriers might also be individuals that adopt a strategy of tolerance (limiting the harm caused by a given parasite burden) rather than resistance (limiting parasite burden) to combat *Mo*. This predisposition for tolerance has a genetic basis and is variable among individuals within a species (Råberg, Graham, & Reed, 2009). Identifying factors associated with carriage status would provide insight into temporal variation in disease dynamics and population response to infection. The ability to determine the likelihood of intermittent versus chronic carriage at an individual or population level would also help minimize the sampling effort required to efficiently identify chronic carriers. Our results indicate the minimum required sampling intensity will vary across populations.

Given the cost of identifying chronic carriers, wildlife managers might question whether complete depopulation followed by repopulation is preferable to selective removal. However, complete depopulation of wildlife is not always feasible, especially in rugged or remote terrain (Courchamp, Chapuis, & Pascal, 2003). Furthermore, while depopulation removes the pathogen along with its host, ensuing reintroductions may fail (Griffith, Scott, Carpenter, & Reed, 1989), with political and economic consequences. Population‐level knowledge also can accumulate and transmit across generations (Jesmer et al., 2018), affecting a population's ability to follow green‐up, avoid predation, and maintain complex migration routes. This knowledge has implications for fitness and is lost with depopulation. While identifying chronic carriers may not be feasible everywhere, selective removal preserves knowledge that may promote population persistence and growth following treatment.

Our study provides experimental evidence for chronic carriage as a mechanism for pathogen (and disease) persistence, and our results may apply to chronic infections across taxa. Chronic carriers enable *Leptospirosa interrogans* to persist through “inter‐epidemic troughs” in California sea lion (*Zalophus californianus*) populations and may play an important role in the spillover of Hendra, Nipah, Marburg, and Ebola from bats to humans and other mammals (Buhnerkempe et al., 2017; Plowright et al., 2016). Test‐and‐remove methods aimed at carriers, such as the one presented in our experiment, are few and have met varied success. For instance, attempts to remove elk testing seropositive for *Brucella abortus* from herds in Wyoming failed to eliminate the pathogen (Scurlock, Edwards, Cornish, & Meadows, 2010). Similarly, culling European badgers (*Meles meles*) in Great Britain counterintuitively increased *Mycobacterium bovis* spillover to nearby cattle herds (Donnelly et al., 2003). An important distinction that may account for the success of our intervention as opposed to these examples is that we removed all chronic carriers. In humans, *Staphylococcus aureus* is similar to *Mo* in that the nares are the primary carriage site and carriers can be classified into chronic, intermittent, and noncarrier categories (Wertheim et al., 2005). Isolating patients that chronically carry *S. aureus* can, but does not always, prevent subsequent infection in at‐risk hospital patients (Peterson & Schora, 2016; Wertheim et al., 2005). One notable difference between *S. aureus* and *Mo* is length of environmental persistence, which may explain inconsistent results of *S. aureus* chronic carrier isolation. *Mo* persists for only minutes in the environment (Citti & Blanchard, 2013), whereas *S. aureus* can persist for months, increasing reinfection opportunities (Neely & Maley, 2000). Environmental persistence and presence of reservoir hosts are important factors to consider when contemplating chronic carrier removal/treatment for a new disease system.

In conclusion, our experiment demonstrates the importance of accounting for individual heterogeneity when determining intervention options to eradicate or reduce the prevalence of persistent infections in a population. Health interventions are often expensive and urgent, and knowledge regarding where to focus limited resources can save money and lives. Focusing resources on identifying and treating chronically carrying individuals can lead to population‐level disease clearance, but the appropriateness of this focus depends on how the disease is spread and maintained within and between populations. As infectious disease outbreaks increase globally (Jones et al., 2008), broadening our understanding of various transmission patterns will be integral to mitigating disease outbreaks and conserving at‐risk wildlife populations.

## CONFLICT OF INTEREST

None declared.

## AUTHORS' CONTRIBUTIONS

C.P.L., J.A.J., T.E.B., E.F.C., and D.P.W. generated ideas and designed methodology. T.J.G. and C.P.L. collected the field data. T.E.B. coordinated laboratory processing of our samples. D.P.W. and T.J.G. analyzed the data. T.J.G. led the writing of the manuscript. All authors contributed critically to the drafts and gave final approval for publication.

## Supporting information

Supinfo

## Data Availability

Data are available at Dryad Digital Repository, https://doi.org/10.5061/dryad.dm7912g.
