# Supplementary material for: Removal of chronic Mycoplasma ovipneumoniae carrier ewes eliminates pneumonia in a bighorn sheep population
Source: Ecol Evol. 2020 Mar 5;10(7):3491–502. doi: 10.1002/ece3.6146 (PMC7141075; doi:10.1002/ece3.6146)
Supplement: Supplementary file 1 — Supinfo [file ECE3-10-3491-s001.docx]

**Supporting Information**

**Table S1:** Testing dates and results for bighorn sheep (*Ovis canadensis*) sampled in the treatment population prior to and shortly after chronic carrier removal, 1 August 2014-8 April 2016. Individuals who always tested positive after their first positive test were considered chronic carriers and removed 13 March 2016. All strain typed positive tests in the treatment population were the resident BHS-058 strain. Antibody presence indicates exposure to *Mycoplasma ovipneumoniae* (*Mo*).

| **Treatment Identifier** | **Sex** | **Age** | **PCR Test Result**^1^ | | | | **Chronic Carrier** | ***Mo* Antibodies**^2^ | **Other Detected Pathogens**^3^ | **Test Dates** | |
| --- | --- | --- | --- | --- | --- | --- | --- | --- | --- | --- | --- |
|  |  |  | **1** | **2** | **3** | **4** |  |  |  |  |  |
| 1 | ♀ | 4+ | - | - | - | - | No | D | *Bt* | 8/1/14, 1/1/15, 10/22/15, 3/1/16 | |
| 2 | ♀ | 4+ | + | + | + | + | Yes | D | *Bt* | 8/6/14, 4/15/15, 10/22/15, 3/13/16 | |
| 3 | ♀ | 4+ | - | - | - |  | No | D | *Bt* | 8/11/14, 11/16/15, 3/13/16 | |
| 4 | ♀ | 4+ | - | - | - |  | No | D |  | 8/26/14, 10/31/14, 9/9/15 | |
| 5 | ♂ | 4+ | - | - | - |  | No | D | *Bt*, *Tp* | 1/15/15, 12/3/15, 3/13/16 | |
| 6 | ♂ | 4+ | - | - | in |  | No | D |  | 1/15/15, 11/5/15, 2/11/16 | |
| 7 | ♀ | 4+ | - | - | - |  | No | D | *LktA* | 1/28/2015, 11/4/15, 3/1/16 | |
| 8 | ♀ | 4+ | - | - | - |  | No | D |  | 1/15/15, 9/15/15, 2/29/16 | |
| 9 | ♀ | 4+ | - | - | - |  | No | D |  | 1/15/15, 10/2/15, 3/7/16 | |
| 10 | ♀ | 0.5 | - | - |  |  | No | D | *Bt*, *Tp* | 3/20/15, 4/8/16 | |
| 11 | ♀ | 0.5 | - | - |  |  | No | D | *Bt*, *Tp* | 3/24/15, 4/8/16 | |
| 12 | ♀ | 1.5 | - | - | - |  | No | D | *Bt* | 4/6/15, 9/11/15, 3/8/16 | |
| 13 | ♂ | 1.5 | - | - |  |  | No | ND | *Ms* | 4/7/15, 4/8/16 | |
| 14 | ♂ | 0.5 | - | - |  |  | No | ND | *Bt*, *Tp*, *Ms* | 4/7/15, 3/13/16 | |
| 15 | ♂ | 2.5 | - | - |  |  | No | ND | *Bt* | 7/28/15, 3/13/16 | |
| 16 | ♂ | 3.5 | - | - |  |  | No | ND | *Bt* | 7/28/15, 3/13/16 | |
| 17 | ♀ | 4+ | - | - |  |  | No | D |  | 7/30/15, 4/8/16 | |
| 18 | ♀ | 4+ | - | - |  |  | No | D | *Bt*, *Tp* | 9/3/15, 3/13/16 | |
| 19 | ♀ | 4+ | + | + | + |  | Yes | D |  | 10/20/14, 10/31/15, 3/13/16 | |
| 20 | ♀ | 4+ | - | - | - |  | No | D | *Tp*, *LktA* | 2/4/15, 4/6/15, 2/22/16 | |
| 21 | ♂ | 0.5 | - |  |  |  | No | in |  | 4/4/2016 | |
| 22 | ♀ | 0.5 | - |  |  |  | No | ND | *Bt, LktA* | 4/4/2016 | |
| 23 | ♀ | 4+ | + | - |  |  | No | D |  | 8/26/14, 5/1/15 | |
| 24 | ♀ | 3.5 | - | - |  |  | No | D |  | | 3/19/15, 10/20/15 |
| ^1^ - = Not Detected, + = Detected, in= Indeterminate; ^2^ D = Detected, ND = Not Detected, in = Indeterminate; ^3^ *Bt* = *Bibersteinia trehalosi*, *Tp* = *Trueperella pyogenes*, *LktA* = Leukotoxigenic *Pasteurella*, *Ms* = *Mannheimia* sp. | | | | | | | | | | | |

**Table S2**: Testing dates and results for bighorn sheep (*Ovis canadensis*) sampled in the control population over the course of the study, 1 January 2016-1 May 2018. Antibody presence indicates exposure to *Mycoplasma ovipneumoniae* (*Mo*). Strain type BHS-058 is the resident strain, and BHS-043 is the introduced strain.

| **Control Identifier** | | **Subherd**^1^ | **Sex** | **PCR Test Result**^2^ | | | | **Chronic Carrier** | **Mo Antibodies**^3^ | **Other Detected Pathogens**^4^ | **Test Dates (Strain Type)** |
| --- | --- | --- | --- | --- | --- | --- | --- | --- | --- | --- | --- |
|  |  |  |  | **1** | **2** | **3** | |  |  |  |  |
| 1 | | HC | ♂ | + |  |  | No | | D | *Lkta* | 4/6/16 (BHS-058) |
| 2 | | RC | ♂ | + | - |  | No | | D | *Tp* | 3/11/16 (BHS-058), 2/5/17 |
| 3 | | RC | ♀ | - | - |  | No | | D | *Tp, Ms* | 3/11/16, 2/5/17 |
| 4 | | RC | ♀ | - | In |  | No | | D | *Bt, Tp* | 3/12/16, 2/4/17 |
| 5 | | RC | ♂ | - | + |  | No | | D | *Tp* | 3/22/16, 2/7/17 (BHS-058) |
| 6 | | RC | ♀ | - | In |  | No | | D | *Tp* | 3/22/16, 2/5/17 |
| 7 | | RC | ♀ | - | - |  | No | | D | *Bt, Tp, Mg* | 3/22/16, 2/5/17 |
| 8 | | RC | ♀ | in | In |  | No | | D | *Bt,Tp, Mh* | 3/22/16, 2/5/17 |
| 9 | | RC | ♂ | - |  |  | No | | D |  | 3/22/16 |
| 10 | | RC | ♂ | + |  |  | No | | D |  | 3/22/16 (BHS-058) |
| 11 | | RC | ♀ | + |  |  | No | | D |  | 3/22/16 (BHS-058) |
| 12 | | RC | ♂ | + | + |  | Yes | | D | *Tp* | 3/22/16 (BHS-058), 2/7/17 (BHS-058) |
| 13 | | RC | ♂ | + |  |  | No | | NT |  | 10/26/16 (BHS-058) |
| 14 | | RC | ♀ | - |  |  | No | | D |  | 11/16/16 |
| 15 | | RC | ♀ | + |  |  | No | | D | *Bt* | 11/17/16 (BHS-058) |
| 16 | | RC | ♂ | - |  |  | No | | D | *Tp* | 2/5/17 |
| 17 | | RC | ♀ | in | + |  | No | | D | *Tp* | 3/11/16, 2/5/17 (BHS-058) |
| 18 | | RC | ♀ | + | + | + | Yes | | D | *Tp* | 3/11/16 (BHS-058), 2/5/17 (BHS-058), 4/17/18 (BHS-058) |
| 19 | | SC | ♀ | - | + | in | No | | D | *Bt, Tp* | 3/13/16, 2/7/17 (BHS-043), 4/6/17 |
| 20 | | SC | ♀ | - |  |  | No | | D | *Bt,Tp* | 3/13/16 |
| 21 | | SC | ♂ | - |  |  | No | | NT |  | 3/22/16 |
| 22 | | SC | ♀ | - | + |  | No | | D | *Tp* | 3/22/16, 2/7/17 (BHS-043) |
| 23 | | SC | ♀ | - | + |  | No | | D | *Bt, Tp* | 3/22/16, 2/7/17 (BHS-043) |
| 24 | | SC | ♀ | - | + |  | No | | D | *Bt, Tp* | 3/22/16, 2/7/17 (BHS-043) |
| 25 | | SC | ♀ | + | + | - | No | | D | *Bt, Tp* | 2/23/16 (neither), 2/7/17 (BHS-043), 1/30/18 |
| 26 | | SC | ♂ | + |  |  | No | | NT |  | 10/6/16 (BHS-058) |
| 27 | | SC | ♂ | + |  |  | No | | D | *Bt, Tp* | 12/13/16 (BHS-043) |
| 28 | | SC | ♀ | + |  |  | No | | D | *Tp* | 12/20/16 (BHS-043) |
| 29 | | SC | ♀ | - | + |  | No | | D | *Bt, Tp* | 2/27/16, 2/7/17 (BHS-043) |
| 30 | | SC | ♂ | - |  |  | No | | D | *Bt* | 2/7/17 |
| 31 | | SC | ♂ | + |  |  | No | | NT | *Tp, Ms* | 2/7/17 (failed) |
| 32 | | SC | ♀ | - |  |  | No | | D | *Tp* | 2/7/17 |
| 33 | | SC | ♀ | in |  |  | No | | D |  | 2/7/17 |
| 34 | | SC | ♀ | - | + |  | No | | D | *Bt, Ms* | 3/7/16, 2/7/17 (BHS-043) |
| 35 | | SC | ♀ | - |  |  | No | | NT |  | 2/2/18 |
| 36 | | SC | ♀ | - | + |  | No | | D | *Bt, Tp* | 3/8/16, 2/7/17 (BHS-043) |
| 37 | | SC | ♀ | - | + |  | No | | D | *Bt* | 3/9/16, 4/13/17 (BHS-043) |
| 38 | | SC | ♂ | - |  |  | No | | D | *Bt* | 3/13/16 |
| 39 | | SC | ♀ | - | - |  | No | | D | *Tp* | 3/13/16, 2/7/17 |
| 40 | | SC | ♀ | + |  |  | No | | D |  | 3/13/16 (BHS-058) |
| 41 | | HC | ♀ | - |  |  | No | | ND |  | 1/22/16 |
| 42 | | HC | ♀ | + |  |  | No | | D | *Bt* | 1/22/16 (BHS-058) |
| 43 | | HC | ♂ | + |  |  | No | | D | *Bt* | 1/27/16 (BHS-058) |
| 44 | | HC | ♀ | in |  |  | No | | D | *Bt, Tp* | 1/27/16 |
| 45 | | RC | ♀ | + |  |  | No | | ND | *Bt* | 2/18/16 (BHS-058) |
| 46 | | RC | ♀ | + |  |  | No | | D | *Bt* | 2/18/16 (NT) |
| 47 | | HC | ♀ | + |  |  | No | | D | *Bt, Tp* | 2/27/16 (BHS-058) |
| 48 | | RC | ♀ | - |  |  | No | | D |  | 3/11/16 |
| 49 | | RC | ♀ | - |  |  | No | | D |  | 3/11/16 |
| 50 | | RC | ♀ | + |  |  | No | | D |  | 3/11/16 (BHS-058) |
| 51 | | RC | ♂ | + |  |  | No | | in |  | 3/11/16 (BHS-058) |
| 52 | | RC | ♂ | + |  |  | No | | NT |  | 3/11/16 (BHS-058) |
| 53 | | RC | ♂ | - |  |  | No | | D |  | 3/11/16 |
| 54 | | RC | ♀ | + |  |  | No | | D |  | 3/11/16 (BHS-058) |
| 55 | | RC | ♀ | - |  |  | No | | D |  | 3/11/16 |
| 56 | | RC | ♀ | in |  |  | No | | D |  | 3/11/16 |
| 57 | | RC | ♀ | + |  |  | No | | D |  | 3/12/16 (BHS-058) |
|  | ^1^ HC = Hill City, RC = Rapid Creek, SC = Spring Creek  ^2^ - = Not Detected, + = Detected, in= Indeterminate; ^3^ D = Detected, ND = Not Detected, NT= Not Tested, in = Indeterminate; ^4^ *LktA* = Leukotoxigenic *Pasteurella*, *Tp* = *Trueperella pyogenes*, *Ms* = *Mannheimia* sp., *Bt* = *Bibersteinia trehalosi*, *Mg* = *Mannheimia glucosida*, *Mh* = *Mannheimia haemolytica* | | | | | | | | | | |

**Table S3**: Testing frequency, results, and dates for bighorn sheep (*Ovis canadensis*) sampled in the treatment population after chronic carrier removal, 9 April 2016-1 May 2018. Antibody presence indicates past exposure to *Mycoplasma ovipneumoniae* (*Mo*).

| Treatment Identifier | PCR Test Result^1^ | | Chronic Carrier | *Mo* Antibodies^2^ | Other Pathogens Detected^3^ | Test Dates |
| --- | --- | --- | --- | --- | --- | --- |
|  | 1 | 2 |  |  |  |  |
| 4 | - |  | No | D | *Tp* | 2/4/17 |
| 5 | - | - | No | D | *Bt, Tp, Ms* | 11/10/16, 2/4/17 |
| 6 | - | in | No | NT | *Tp* | 11/28/16, 2/8/17 |
| 7 | - |  | No | in |  | 2/4/17 |
| 8 | - |  | No | ND |  | 2/4/17 |
| 9 | - | in | No | ND | *Ms, Mh* | 11/1/16, 2/4/17 |
| 10 | - |  | No | D |  | 2/6/17 |
| 11 | - | - | No | D | *Bt* | 10/15/16, 2/4/17 |
| 12 | - |  | No | ND | *Bt, Tp* | 2/6/17 |
| 13 | - |  | No | NT |  | 2/6/17 |
| 15 | in | - | No | D |  | 8/5/16, 2/8/17 |
| 16 | in |  | No | D | *Ms* | 2/4/17 |
| 17 | - |  | No | NT |  | 2/4/17 |
| 18 | - |  | No | D |  | 2/4/17 |
| 20 | - |  | No | D |  | 10/16/16, 2/4/17 |
| 21 | - |  | No | in |  | 2/4/17 |
| 22 | - |  | No | ND |  | 2/4/17 |
| 25 | in |  | No | ND | *Bt, Ms* | 2/8/17 |
| 26 | - |  | No | NT | *Bt* | 2/6/17 |
| 27 | - |  | No | NT |  | 2/6/17 |
| 28 | - | in | No | ND |  | 8/8/16, 2/8/17 |
| 29 | - | in | No | ND |  | 8/8/16, 2/8/17 |
| 30 | in | in | No | ND | *Bt* | 9/20/16, 2/4/17 |
| 31 | - | - | No | D | *Bt* | 9/21/16, 2/4/17 |
| 32 | - |  | No | NT | *Ps* | 2/6/17 |
| 33 | - |  | No | D | *Bt* | 9/18/17 |
| ^1^ - = Not Detected, + = Detected, in = Indeterminate; ^2^ D = Detected, ND = Not Detected, NT= Not Tested, in = Indeterminate ; ^3^ *Tp* = *Trueperella pyogenes*, *Bt* = *Bibersteinia trehalosi*, *Ms* = *Mannheimia* sp, *Mh* = *Mannheimia haemolytica*, *Ps* = *Pastuerella* sp. | | | | | | |

**Table S4**: Cause of death probabilities assigned to all a.) adults and b.) lambs included in the survival analysis. For analysis, we converted these probabilities into vectors that summed to one across cause-specific categories for each individual that died.

| a.) | Treatment Adult Identifier | | Human Caused | Other | Pneumonia | Predation | b.) | Treatment Lamb Identifier | Year | Other | Pneumonia | Predation |
| --- | --- | --- | --- | --- | --- | --- | --- | --- | --- | --- | --- | --- |
|  | | 1 |  |  |  | 100% |  | 1 | 2016 | 5% | 95% |  |
|  | | 3 |  | 5% | 5% | 90% |  | 2 | 2017 |  | 100% |  |
|  | | 6 |  |  |  | 100% |  | 3 | 2017 |  | 100% |  |
|  | | 7 |  | 100% |  |  |  | 4 | 2017 | 100% |  |  |
|  | | 14 |  |  |  | 100% |  | 5 | 2017 |  | 100% |  |
|  | |  |  |  |  |  |  | 6 | 2017 | 100% |  |  |
|  | |  |  |  |  |  |  | 7 | 2017 |  | 100% |  |
|  | |  |  |  |  |  |  |  |  |  |  |  |
|  | |  |  |  |  |  |  |  |  |  |  |  |
|  | | Control Adult Identifier | Human Caused | Other | Pneumonia | Predation |  | Control Lamb Identifier | Year | Other | Pneumonia | Predation |
|  | |  |  |  |  |  |  |  |  |  |  |  |
|  | | 1 |  | 40% | 60% |  |  | 1 | 2016 | 60% | 40% |  |
|  | | 9 | 100% |  |  |  |  | 2 | 2016 | 100% |  |  |
|  | | 12 |  | 40% | 60% |  |  | 3 | 2016 |  | 100% |  |
|  | | 20 |  | 5% | 95% |  |  | 4 | 2016 | 10% |  | 90% |
|  | | 21 |  |  | 100% |  |  | 5 | 2016 |  | 100% |  |
|  | | 24 |  | 90% |  | 10% |  | 6 | 2016 | 100% |  |  |
|  | | 28 | 25% |  | 75% |  |  | 7 | 2016 |  | 100% |  |
|  | | 41 | 100% |  |  |  |  | 8 | 2016 | 100% |  |  |
|  | | 42 | 100% |  |  |  |  | 9 | 2016 | 100% |  |  |
|  | | 43 |  | 10% | 90% |  |  | 10 | 2016 | 34% | 33% | 33% |
|  | | 45 | 100% |  |  |  |  | 11 | 2016 |  | 100% |  |
|  | | 46 |  |  | 25% | 75% |  | 12 | 2017 | 51% | 49% |  |
|  | | 47 | 25% |  | 75% |  |  | 13 | 2017 |  |  | 100% |
|  | | 49 |  |  | 100% |  |  | 14 | 2017 | 100% |  |  |
|  | | 50 |  | 70% | 30% |  |  | 15 | 2017 |  | 100% |  |
|  | | 52 |  | 10% | 90% |  |  | 16 | 2017 |  | 100% |  |
|  | | 55 |  | 5% | 95% |  |  | 17 | 2017 |  | 40% | 60% |
|  | |  |  |  |  |  |  | 18 | 2017 |  | 100% |  |

**Table S5**: Descriptions and rankings of all models tested to explain log unit cumulative hazard [ln(Λ_i,j_)] for each individual adult bighorn sheep (*Ovis canadensis*), 13 March 2016 – 1 May 2018. Ranking is based upon Watanabe-Akaike Information Criteria (WAIC) and is reported with ΔWAIC (difference in WAIC between top model and model being compared) and *w_i_* (WAIC weight). γ is baseline log unit cumulative hazard rate, β_treatment_ is the effect of the treatment (whether an individual was in the treatment or control herd), β_year_ is the effect of year, β_strain_ is the effect of strain type, β_age_ is the effect of age, β_sex_ is the effect of sex, β_positive test_ is the effect of having at least one positive test, and ρ_j_ is the effect of a given week (j) with a random walk prior for temporal smoothing across estimates.

| **Model Description** | **Effects** | **WAIC** | **ΔWAIC** | ***w_i_*** |
| --- | --- | --- | --- | --- |
| 1) Hazard varied by treatment | γ, β_treatment_, ρ_j_ | 344.5 | 0 | 0.31 |
| 2) Hazard varied by treatment and sex | γ, β_treatment_, β_sex_, ρ_j_ | 346.0 | 1.5 | 0.15 |
| 3) Hazard varied by treatment and strain type | γ, β_treatment_, β_strain_, ρ_j_ | 346.5 | 2.0 | 0.12 |
| 4) Hazard varied by strain type | γ, β_strain_, ρ_j_ | 346.6 | 2.1 | 0.11 |
| 5) Hazard varied by treatment and positive test | γ, β_treatment_, β_positive test_, ρ_j_ | 346.8 | 2.3 | 0.10 |
| 6) Hazard varied by treatment and year | γ, β_treatment_, β_year_, ρ_j_ | 347.1 | 2.6 | 0.09 |
| 7) Hazard varied by treatment and age | γ, β_treatment_, β_age_, ρ_j_ | 348.3 | 3.8 | 0.05 |
| 8) Hazard varied by treatment, year, and sex | γ, β_treatment,_ β_year_, β_sex_, ρ_j_ | 348.7 | 4.2 | 0.04 |
| 9) Hazard varied by treatment, age, and sex | γ, β_treatment_, β_age_, β_sex_, ρ_j_ | 349.3 | 4.8 | 0.03 |
| 10) Global Model | γ, β_treatment_, β_year_, β_age_, β_sex_, β_positive test_, ρ_j_ | 351.0 | 6.5 | 0.01 |
| 11) Base Model | γ, β_treatment_, ρ_j_ | 356.5 | 12.0 | 0.00 |

**Table S6**: Descriptions and rankings of all models tested to explain log unit cumulative hazard [ln(Λ_i,j_)] for each individual bighorn sheep (*Ovis canadensis*) lamb, 1 May 2016 – 1 November 2016 and 1 May 2017 – 1 November 2017. Ranking is based upon Watanabe-Akaike Information Criteria (WAIC) and is reported with ΔWAIC (difference in WAIC between top model and model being compared) and *w_i_* (WAIC weight). γ is baseline log unit cumulative hazard rate, β_treatment_ is the effect of the treatment (whether a lamb was in the treatment or control herd), β_year_ is the effect of year, β_strain_ is the effect of strain type, β_sex_ is the effect of sex, β_birth_ _timing_ is the effect of birth timing, β_birth_ _weight_ is the effect of birth weight, and ρ_j_ is the effect of a given day (j) with a random walk prior for temporal smoothing across estimates.

| **Model Description** | **Effects** | **WAIC** | **ΔWAIC** | ***w_i_*** |
| --- | --- | --- | --- | --- |
| 1) Hazard varied by treatment and year | γ, β_treatment_, β_year_, ρ_j_ | 310.5 | 0 | 0.64 |
| 2) Hazard varied by treatment, year, and sex | γ, β_treatment_, β_year_, β_sex_, ρ_j_ | 312.8 | 2.3 | 0.20 |
| 3) Hazard varied by treatment | γ, β_treatment_ | 314.1 | 3.6 | 0.11 |
| 4) Hazard varied by treatment and strain type | γ, β_treatment_, β_strain_, ρ_j_ | 315.7 | 5.2 | 0.05 |
| 5) Base Model | γ, ρ_j_ | 321.9 | 11.4 | 0.00 |
| 6) Hazard varied by strain type | γ, β_strain_, ρ_j_ | 322.5 | 12.0 | 0.00 |
| 7) Hazard varied by strain type and year | γ, β_treatment_, β_strain_, ρ_j_ | 324.1 | 13.6 | 0.00 |
| 8) Hazard varied by treatment, year, and birth timing | γ, β_treatment_, β_year_, β_birth timing_, ρ_j_ | 417.7 | 107.2 | 0.00 |
| 9) Hazard varied by treatment, year, sex, and birth weight | γ, β_treatment_, β_year_, β_birth weight_, ρ_j_ | 492.3 | 181.8 | 0.00 |
| 10) Global Model | γ, β_treatment_, β_year_, β_sex_, β_birth timing_, β_birth weight_, ρ_j_ | 603.9 | 293.4 | 0.00 |

**
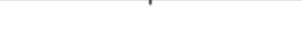

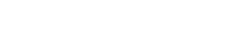

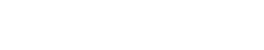
Figure S1**: Overall log hazard for an adult bighorn sheep (*Ovis canadensis*) in our study (γ), 13 March 2016-1 May 2018. Plot is based on our top model, ln(Λ_i,j_) = γ +β_treatment_ x treatment_i_ + ρ_j_, where ln(Λ_i,j_) is the unit log cumulative hazard for the i^th^ individual in the j^th^ week, β_treatment_ is the effect of being in the treatment population, treatment_i_ denotes whether individual i was in the treatment population, and ρ_j_ is the effect of a given week (j), which is temporally smoothed via a conditional intrinsic autoregressive random walk prior. Brackets indicate peak periods for each cause specific mortality source, and 95% credible intervals are shown in pink and aqua. Overlaps between credible intervals are represented in blue-grey. Note that pneumonia mortality only occurred in the control population.


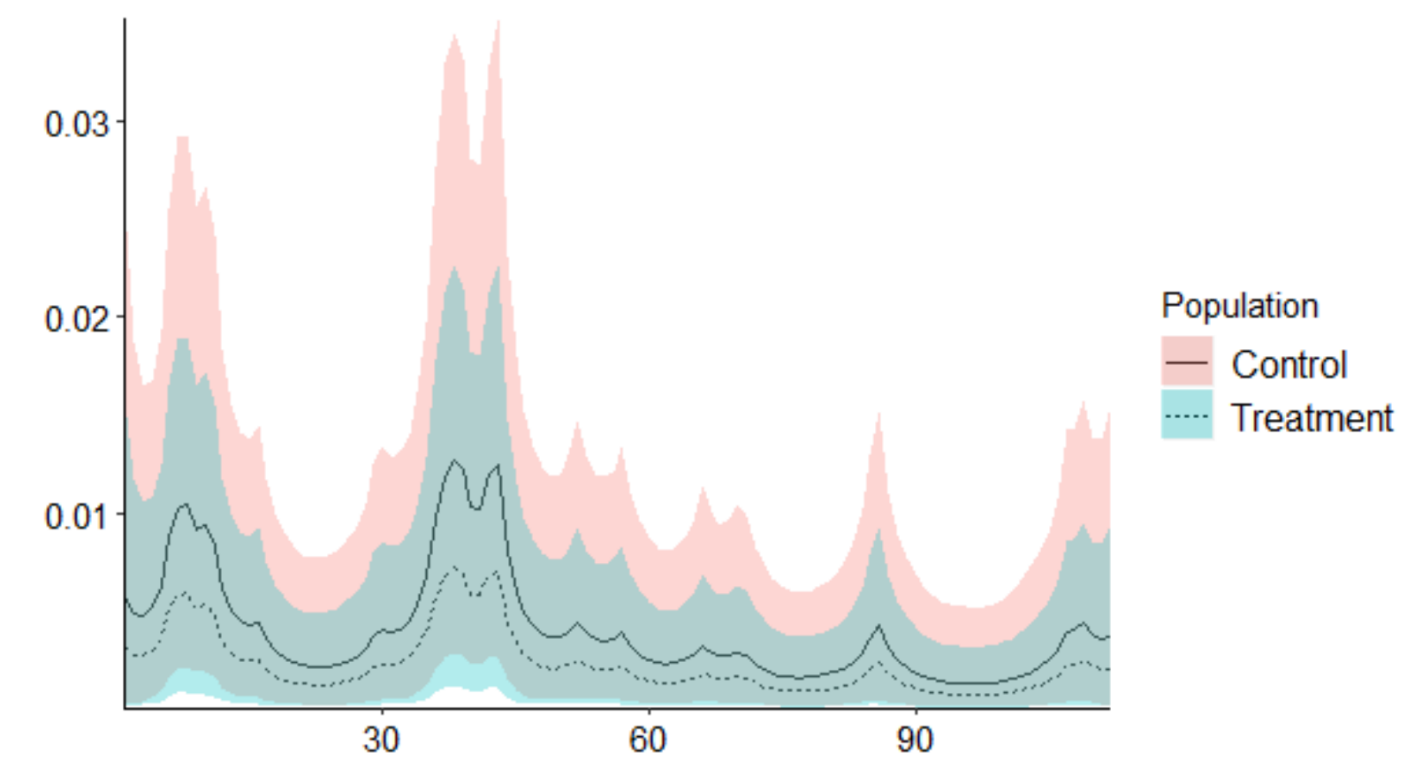


Predation

Other

Month and Year

Pneumonia and Human

May 2017

October 2016

December 2017

log Hazard

0.01

0.01

0.01

**Figure S2**: Overall log hazard for the first six months of a bighorn sheep (*Ovis canadensis*) lamb’s life in Rapid City (γ) during our study (2016 and 2017). Plot is based on our top model, ln(Λ_i,j_) = γ + β_treatment_ x treatment_i_ + β_year_ x year_i_ + ρ_j_, where ln(Λ_i,j_) is the unit log cumulative hazard for the ith individual in the jth day, β_treatment_ is the effect of being in the treatment population, treatment_i_ denotes whether individual i was in the treatment population, β_year_ is the effect of 2017, year_i_ denotes whether individual i was born in 2017, and ρ_j_ is the effect of a given day (j), which is temporally smoothed via a conditional autoregressive random walk prior. Peak periods for each cause specific mortality source are indicated by brackets, and 95% credible intervals are shown in pink and aqua. Overlaps between credible intervals are in represented in blue-grey. For resolution, X-axis begins on day 7 to remove large CI associated due to low sample sizes early in the year; the first lamb died on day 18. Note that pneumonia mortality only occurred in the control population.


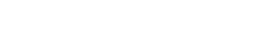

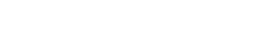


Other Causes

Pneumonia

Predation


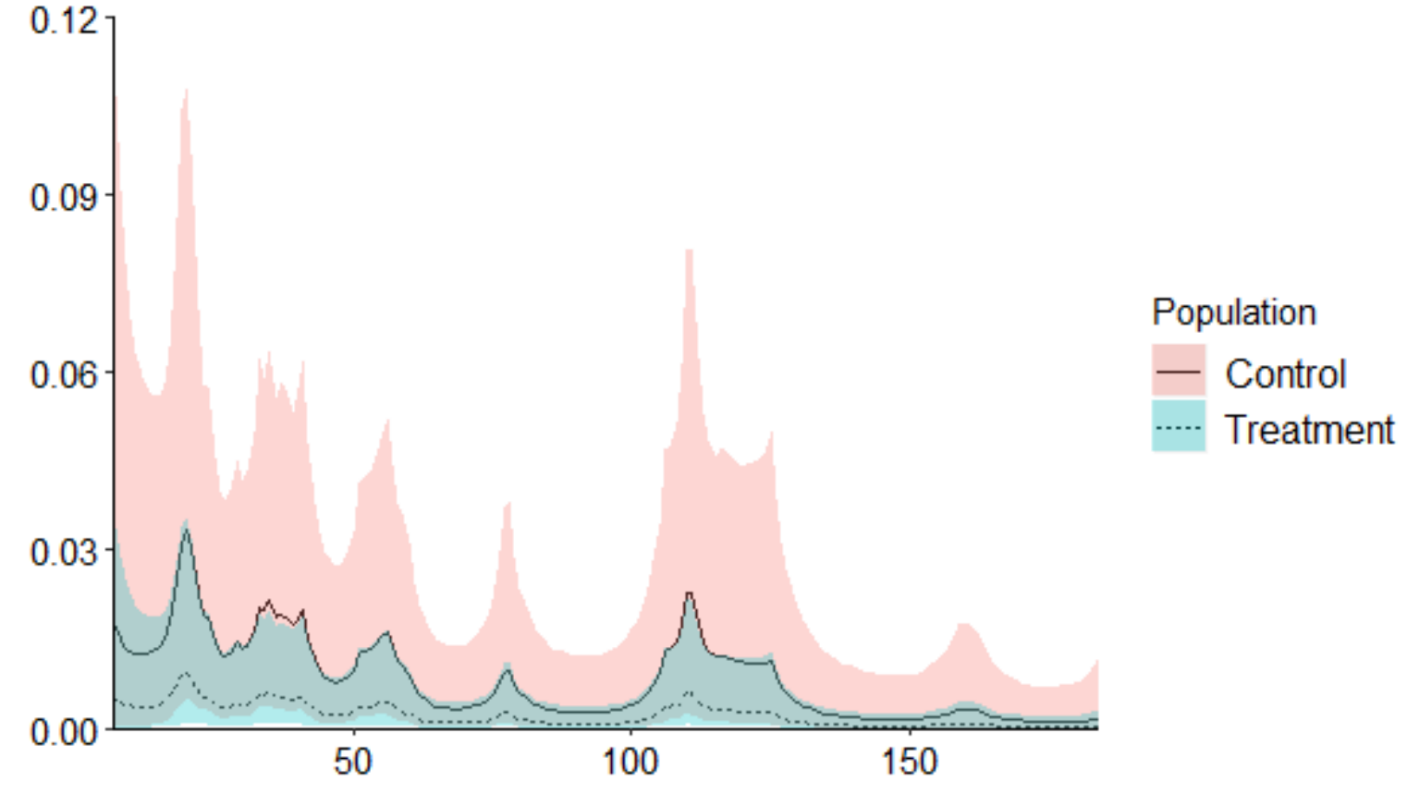


June

August

October

0.06

0.09

0.00

0.03

0.12

Month

log Hazard
